# Thermographic Analysis of the Cooling Dynamics of Liquid Metal Droplets

**DOI:** 10.1021/acsomega.6c05488

**Published:** 2026-09-17

**Authors:** Argelia Balbuena Ortega, Luckenson Augustin, Emiliano Hernández-Figueroa, Sergio Cuevas, Jesús Antonio del Río Portilla

**Affiliations:** † Instituto de Energías Renovables, Universidad Nacional Autónoma de México, Av. Xochicalco S/N, 62580 Temixco, Morelos, Mexico; ‡ Secretaría de Ciencia, Humanidades, Tecnología e Innovación, Av. Insurgentes Sur 1582, Col. Crédito Constructor, 03940 Benito Juárez, Ciudad de México, Mexico

## Abstract

This work presents an experimental study of the thermal dynamics of cooling galinstan liquid metal droplets using thermographic and optical imaging techniques. Galinstan droplets were subjected to controlled cooling on a Peltier-based system with their temperature and morphological evolution recorded simultaneously. Thermography enabled the detection of transient thermal phenomena and localized phase transitions, including supercooling and dendrite formation. The analysis revealed complex, nonequilibrium behaviors, such as metastable thermal and morphological behaviors with coupled variations in temperature and droplet volume that may be consistent with transient or heterogeneous intermediate states. A normalization procedure based on droplet volume enabled comparison across samples, revealing characteristic features in the cooling and heating cycles despite differences in the droplet morphology. Furthermore, directional contact angle measurements using image processing revealed significant variations in wetting behavior throughout the cooling process, showing a gradual reduction in contact angles during intermediate stages, followed by a partial recovery upon rapid phase transformation. Crucially, unlike previous studies that rely on ultrahigh-vacuum chambers and highly specialized equipment, this entire diagnostic methodology was developed under ambient conditions. This makes the approach significantly more practical and optimal for translating liquid metals to real-world applications. The methodology is simple and highly sensitive, providing a noninvasive approach to studying phase transitions in thermally dynamic systems.

## Graphical abstract

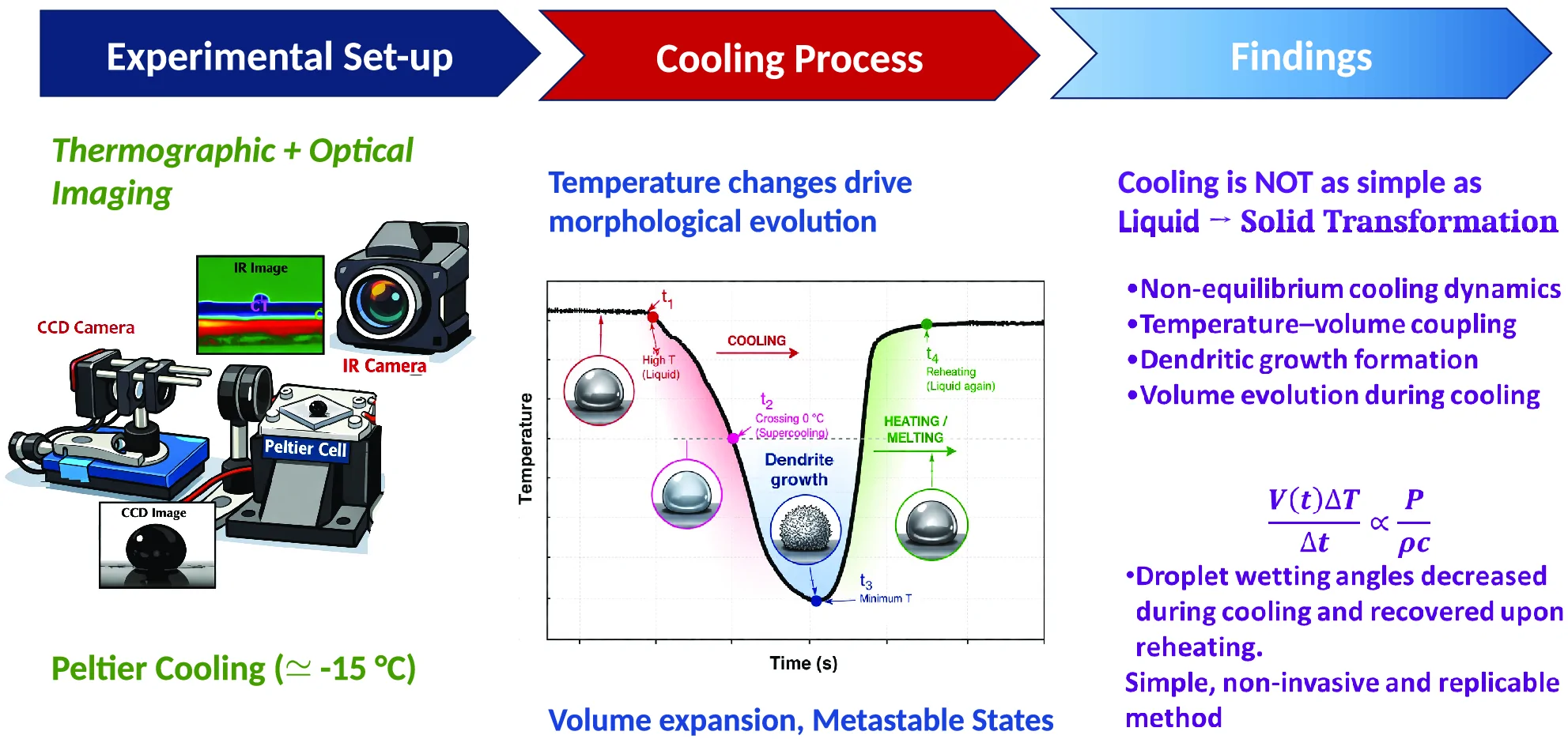

## 1 Introduction

Although seemingly simple, droplets represent a captivating subject of scientific inquiry that spans multiple disciplines. The study of droplets, known as droplet science or droplet dynamics, investigates the complex behavior and properties of liquid droplets across the microscopic to the macroscopic scale. This interdisciplinary field spans physics, chemistry, engineering, and biology offering a wealth of phenomena to explore and understand. Over the years, key droplet behaviors, such as coalescence,^1–3^ breakup, and interactions with solid surfaces,^4–6^ have been studied. These phenomena are central to numerous applications, ranging from industrial manufacturing and thermal management to environmental monitoring and medical diagnostics.^7–10^ By examining how droplets behave, scientists gain insight into the fundamental principles that govern liquid systems—insights that often translate into practical innovations. The study of droplets in heat transfer processes can provide insight into the behavior of fluids when they come into contact with surfaces at different temperatures. In this work, we are interested in the interaction between cold surfaces and droplets of the liquid metal Galinstan.

Liquid metals possess remarkable mechanical properties such as high surface tension and low viscosity. Furthermore, they exhibit the best combination of conductivity and deformability among any known material. This unusual combination has positioned gallium-based liquid metals as promising alternatives for enabling unconventional functions. Due to their high electrical conductivity, liquid metals such as eutectic gallium–indium (EGaIn) or gallium–indium-tin (galinstan) are considered the best candidates for implementing reconfigurable electronics in microfluidic devices, also known as soft robotics.^11,12^ Unlike mercury, these materials have low toxicity and virtually no vapor pressure, making their safe use in laboratories and technological applications feasible. Over the past two decades, these materials have found successful applications in numerous technologically significant areas, such as sensors,^13,14^ chip cooling systems,^15,16^ reconfigurable antennas,^17^ optical switches,^18,19^ technologies with biomedical applications,^20^ and three-dimensional (3D) manufacturing techniques,^21,22^ among others.

Galinstan has been found to present “intriguing” and sometimes inconsistent physical properties. In particular, as water, Galinstan exhibits a density anomaly during freezing, which can be used to generate thermally switchable electronics.^23^ In fact, Galinstan’s temperature-dependent behavior has been studied at higher temperatures around 550 K, showing temperature-dependent heat capacity and liquid–liquid phase transitions.^24^

Oxide-free liquid metals have commonly been classified as Newtonian fluids, although their characterization has been limited. However, it has been known since the last century that molten metals at high temperatures under steady shear stress exhibit non-Newtonian behavior.^25^ More recent studies have re-examined the understanding of liquid metal flow, finding that under certain conditions, they exhibit non-Newtonian characteristics with shear thinning and time-independent flow behavior.^26,27^ It has also been found that under small applied stresses, liquid gallium alloys exhibit elastic properties. In the past decades, it was discovered that gallium and its alloys exhibit viscoelastic behavior, primarily due to the formation of a thin oxide layer on their surface when exposed to ambient air or water.^28,29^ Under these conditions, the mechanical properties of liquid metals are much more complex than those of a Newtonian fluid. The oxide layer restricts the free movement of the liquid metal to a certain extent; on one hand, it is rigid enough to provide mechanical stability to small droplets, and on the other hand, it is flexible enough to allow their deformation. These properties are useful in both microelectronics and microfluidics. Moreover, different interfacial properties of these materials such as adherence, wettability and electrical resistance are determined by this oxide layer with nanoscale thickness.^30^ In fact, it has been pointed out that the unusual behavior of liquid gallium and its alloys, in particular, is due to the thin, solid, native oxide “skin” that forms rapidly on the metal’s surface in the presence of oxygen. For instance, Joshipura et al.^31^ explored how this oxide skin renders conventional contact angle measurements for gallium-based liquid metals nearly useless, noting that the oxide’s mechanical properties cause enormous hysteresis, keep advancing angles consistently high, and result in the droplet base expanding and pinning the oxide to the substrate during receding measurements.

While the response to mechanical interactions of Galinstan and other gallium-based liquid metals has been widely studied (*v.e.* ^26–29,32,33^ ), their response to thermal interactions has received much less attention. In particular, the bulk solidification and melting of Galinstan have been investigated using rheology, X-ray scattering, and differential scanning calorimetry by Koh et al.^34^ They found that Galinstan retains “liquid-like” behavior down to −10 °C, but at lower temperatures solidification depends on confinement, and significant heating is required to restore fluidity. In turn, Joshipura et al.^35^ investigated how the atomically smooth native oxide layer promotes supercooling in bulk liquid gallium, preventing the metal from contacting heterogeneous nucleation sites and allowing it to remain liquid at temperatures significantly below its melting point. This finding was confirmed through differential scanning calorimeter (DSC) and macroscopic observations. In particular supercooling in Galinstan appears as low as −10 °C to −21 °C before finally crystallizing. The extent of supercooling is highly dependent on factors such as the alloy’s purity, thermal history, and the presence of the oxide skin, which can sometimes suppress heterogeneous nucleation.^23^

In this work, we used thermography and optical visualization techniques to characterize the cooling process of galinstan droplets. Thermography is a noncontact imaging technique that detects infrared radiation emitted by a surface to generate a temperature map. It has been used, for example, to analyze free-surface swirling flows in electromagnetically driven electrolyte layers.^36^ Applied to the study of droplets, thermography allows us to visualize temperature variations on their surfaces and, thus, their heat distribution. It also allows us to identify localized thermal events and observe the dynamic temperature changes over time. Using image processing and information obtained from the thermal imaging camera, we identified transient thermal phenomena, including points related to the droplet’s phase change in the cooling process, observing supercooling and dendrite formation. Moreover, using image processing, significant variations in the wetting behavior during the cooling process were found through directional contact angle measurements.

## 2 Experimental Process and Image Analysis

This section outlines the experimental setup and methodology employed to study the cooling behavior of liquid metal (LM) droplets during freezing. We used the eutectic alloy Galinstan Ga(68%)­In(20%)­Sn(12%) whose mass density, kinematic viscosity, and electrical conductivity at room temperature are 6360 kg/m3, 3.4 × 10–7 m2/s, and 3.46 × 106 S/m, respectively. The experimental configuration used in this study is illustrated in Figure 1a. It consists of two optical imaging systems (a thermographic camera and a visible-spectrum system) and a cooling system. Our cooling system consists of a heatsink, a fan, an aluminum plate, and three Peltier cells. The entire system is constructed in layers. The first layer is a Peltier cell, onto which the droplet is placed. The second layer consists of an aluminum plate, while the third layer comprises two Peltier cells. The fourth and final layers are the heatsink and the fan, respectively. This arrangement enables us to achieve temperatures between −12 °C and −20 °C without additional thermal insulation. The thermal imaging system is a FLIR x6540sc, equipped with a 50 mm f/2.0 MWIR USL lens. It features an Indium Antimonide (InSb) detector with a spatial resolution of 640 × 512 pixels and operates within the 1.5–5.5 μm midwave infrared range, capable of detecting temperature variations as small as 20mK. The visible imaging system is based on a custom-built 4f microscope setup, using lenses with focal lengths of *f*_1_ = 5 cm and *f*_2_ = 3 cm, yielding an optical magnification of *M* = 1.6. A Thorlabs CCD camera is integrated into this system to enable high-resolution imaging of droplets during cooling. Since the emissivity of the liquid metal droplet may vary during cooling due to surface oxidation and changes in surface condition, a representative emissivity value of 0.3 was used throughout the measurements.^37^ To minimize errors associated with reflected infrared radiation, the optical path between the camera and the droplet was enclosed to reduce reflections from the surrounding environment. Additionally, the temperature difference between the droplet and the cooling stage was independently monitored using the temperature trace of the Peltier cell, providing a reference for the thermal evolution observed in the infrared measurements.

**Figure 1:**
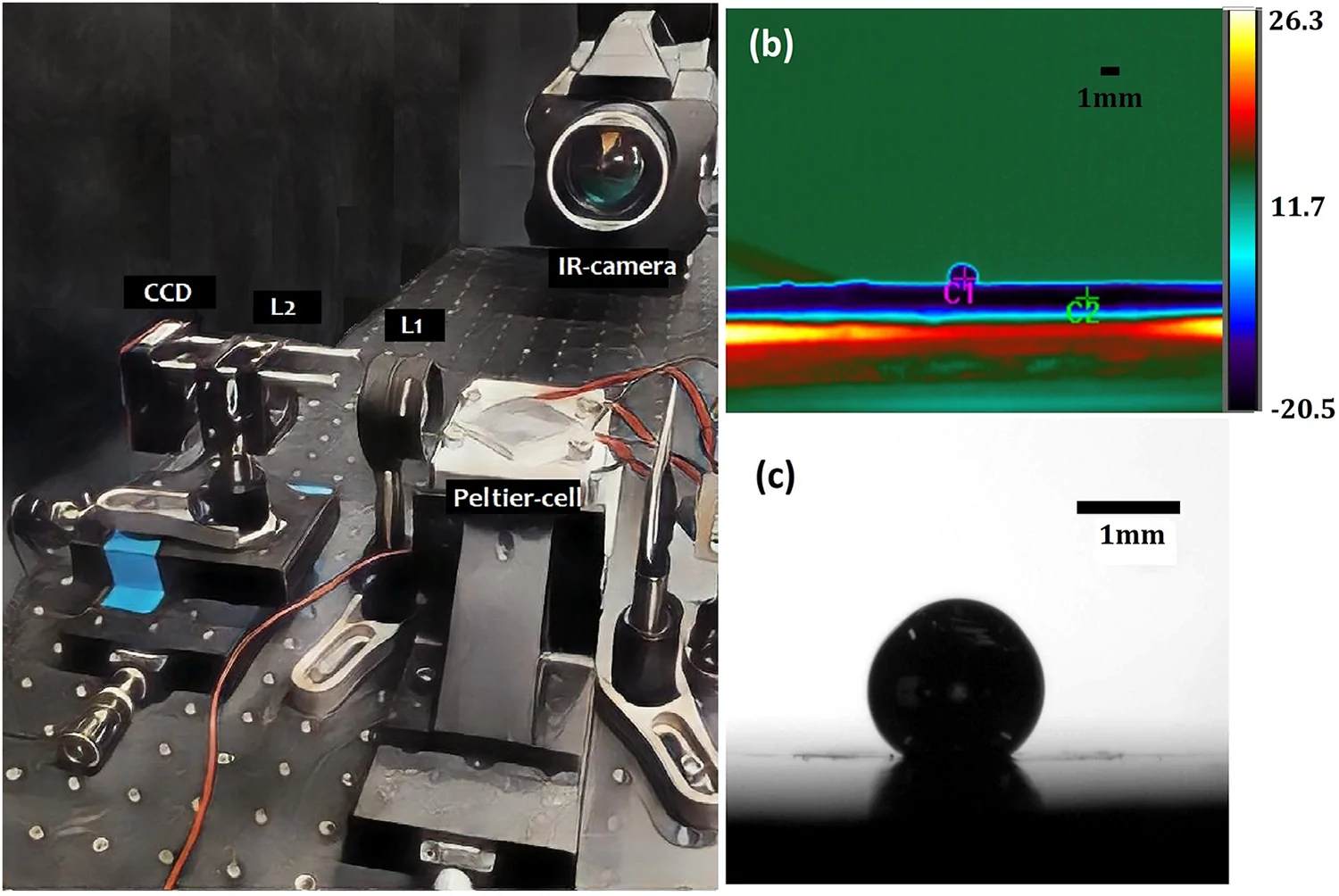
(a) Experimental setup consists of a Peltier cell, and two visualization systems. The first one is a Thermographic camera and the second one is formed by two lenses (L1 = 5 cm and L2 = 3 cm) and a CCD camera. (b) Temperature was tracked over time using indicators C1, positioned at the drop’s center, and C2 located on the Peltier cell. (c) Droplet’s image taken by the CCD camera.

The experimental methodology was as follows: We simultaneously started video recording on the thermographic and CCD cameras. A few seconds later, in the first experiment, a LM droplet was deposited on the Peltier surface (which has an aluminum layer), and the cooling system was immediately activated. A liquid metal droplet was drawn into a pipet from a glass container in which Galinstan was covered with a layer of sulfuric acid solution to prevent oxidation. The experiments were conducted under ambient relative humidity conditions ranging from approximately 25% to 35%, a minor value of humidity would imply a lack of crystallization. Dendrites can be observed between −10 and −20 °C. A total of 70 independent experimental runs were conducted under similar environmental conditions using different initial volumes. Figure 2 shows a probability distribution of dendrite formation as a function of droplet volume. The experimental results demonstrate a clear, volume-dependent threshold for dendrite formation. At lower volumes (1–4 μL and 5–8 μL), the probability of dendrite growth remains consistently high, fluctuating between 80% and 78%. However, a sharp decline to 39% occurs when the volume increases to the 9–12 μL range. This behavior may be influenced by heat transfer constraints associated with droplet size. Larger droplets possess a significantly greater thermal mass and a lower surface-area-to-volume ratio, which could hinder rapid and uniform heat transfer throughout the droplet. This increases the internal thermal resistance, making rapid and uniform heat transfer harder to achieve throughout the entire volume of the droplet. The manual deposition process introduces inherent experimental variability. Due to the high viscoelasticity of the liquid metal, precise volume control via standard pipetting was challenging, resulting in the observed volume spread. The data confirm that this procedural variance directly impacts the robustness of the experiment, establishing that smaller droplet volumes are required to ensure reproducible dendrite formation under these specific cooling configurations. All droplets within this range exhibited similar thermal behavior. We also observed that small changes in droplet morphology could affect the onset of crystallization and the appearance of dendritic structures.

**Figure 2:**
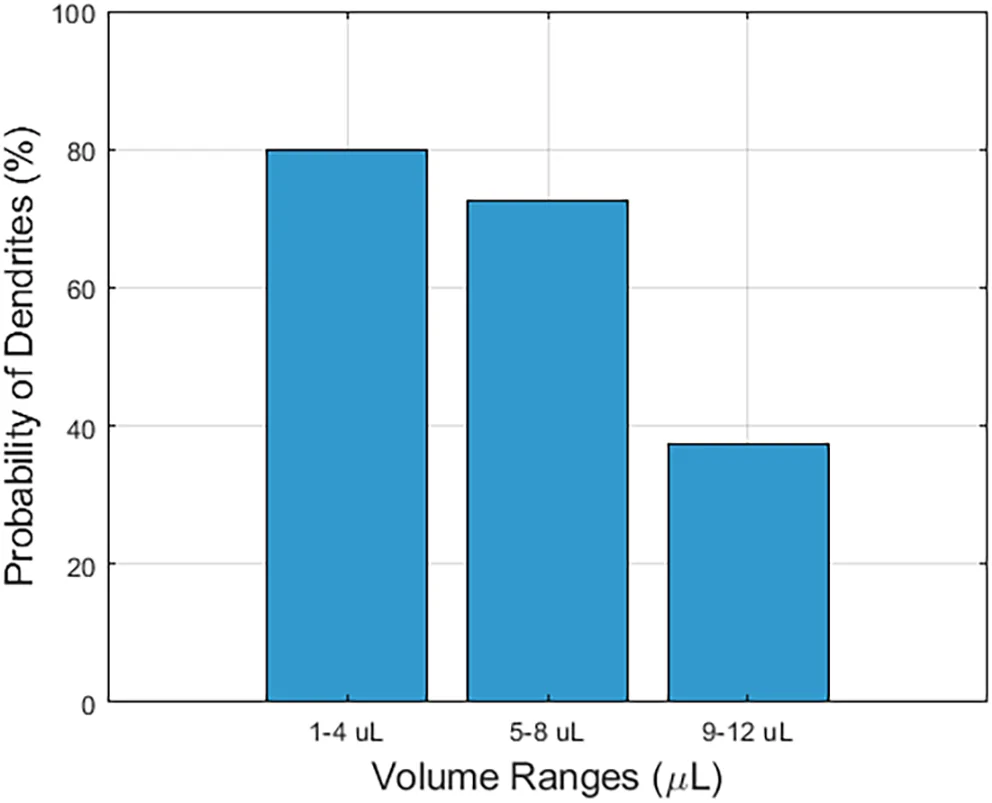
Dendrite formation probability (%) as a function of experimental volume ranges.

In this work, we show the results for droplets that volume varies from 3.45 to 4 μL. The reason is that droplets of this size are large enough to be accurately resolved by the thermographic camera, yet small enough to be conveniently analyzed using the CCD camera. As will be shown later, the size evolves over time. Both thermographic and optical videos captured the cooling process, which began at an initial temperature of approximately 17 °C and continued until dendritic structures appeared on the droplet’s surface. We chose this moment because it is indicative of nucleation, which tells us that the liquid metal could have partially nucleated regions at that temperature. The cooling system was then turned off and allowed to reheat until room temperature was reached. Thermographic videos were recorded at 99 frames per second and analyzed using ResearchIR 4 software. In each video, we tracked the temperature over time using two indicators, as shown in Figure 1b: One (C1) positioned at the droplet’s center and the other (C2) on the Peltier cell. The Peltier indicator served as a reference for the droplet’s actual temperature and marked the start of data collection, i.e., when the cooling system was activated. Figure 1c shows the image of the drop taken by the CCD camera, and the video was recorded with the Thorlabs scientific camera software. The temperature values reported in this work correspond to the infrared temperature measured on the droplet surface using the thermographic camera.

Figure 3 shows a typical example of the thermal evolution of the Galinstan droplet. Figure 3a shows optical and thermal images of the droplet at different times, from the initial temperature to the final reheating stage; the shape, especially the apparent volume of the droplet, changes with the state of cooling. In Figure 3b, the temperature evolution recorded during these measurements is shown. Initially, the system remained in a quasi-steady state (*t*_1_). Activation of the cooling mechanism at *t*_2_ produces a rapid temperature decrease, indicating enhanced heat extraction dominated by forced convection or phase change effects. The minimum temperature at *t*_3_ corresponds to the maximum cooling intensity. After cooling is reduced or terminated, the temperature increases sharply and progressively approaches a new quasi-steady state (*t*_4_) governed by conductive and convective heat transfer. The asymmetry between cooling and reheating slopes reflects the different heat transfer mechanisms controlling each stage of the transient response.

**Figure 3:**
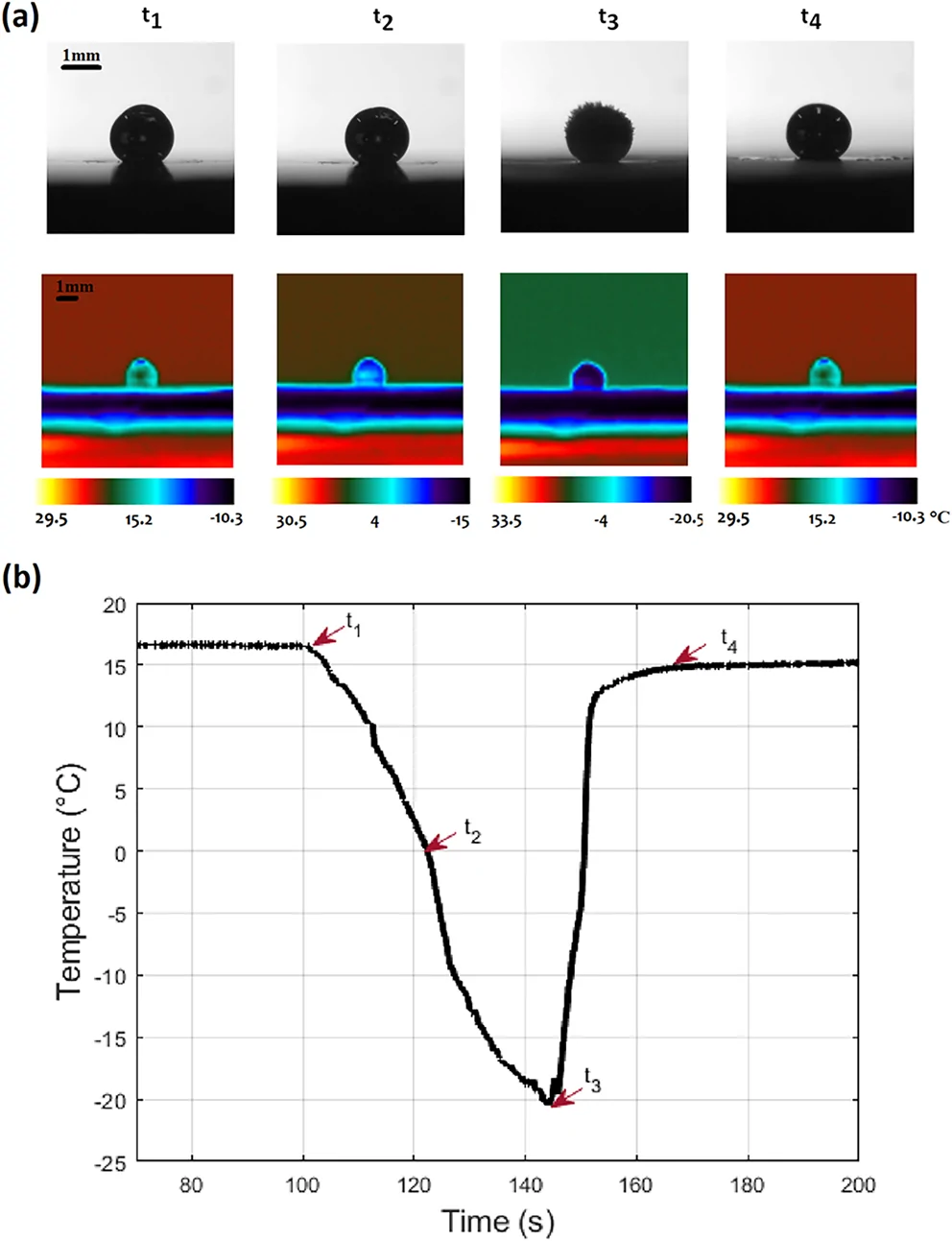
(a) Sequential images of a LM droplet during a freeze–thaw cycle captured at four characteristic times: *t*_1_ (initial liquid state), *t*_2_ (beginning of freezing), *t*_3_ (fully frozen state), and *t*_4_ (thawed and returned to liquid state). The thermographic images reveal the progressive cooling of the droplet and substrate; units are °C. (b) Corresponding temperature profile of the droplet over time, showing rapid cooling, supercooling before nucleation, a sharp drop to the freezing point, and subsequent rewarming. The arrows indicate the times matching the snapshots in (a).

## 3 Results and Discussion

Besides the substrate temperature evolution profile, Figure 4 shows the temperature behavior of three different liquid metal droplets under the cooling process, with temperature plotted against time. Temperatures before and after the cooling process are shown. Initially, all curves exhibit a stable temperature of around 16–17 °C, indicating thermal equilibrium before the onset of cooling. A rapid cooling phase begins around 105 s, in which the temperature of each droplet decreases significantly. The droplets were cooled at different rates and reached different minimum temperatures, the differences being associated with the volume and droplet morphology. Even if the droplet was deposited with a pipet, the liquid metal could bring a small portion of acid from the container, which may have affected the shape, amount, and material composition of the liquid metal. Between 120 and 145 s, all curves display subtle features such as inflection points or shoulders—regions where the slope of the droplet temperature changes. These features could be connected with transitional states of the liquid metal droplets, as well as the formation of metastable intermediate structures,[Fn fn1] rather than a straightforward solidification. In fact, similar thermal features have been observed in studies of freezing water droplets, where simultaneous optical imaging demonstrated that temperature excursions were associated with recalescence, partial crystallization, and the propagation of phase-transition fronts.^38^ In those experiments, abrupt temperature changes coincided with observable structural transformations of the droplet. In the present study, these intermediate states could be associated with the complex phase behavior that is not limited to conventional liquid-to-solid transitions. However, this interpretation cannot be confirmed based on the current measurements alone, and it requires further analysis. There is a region before the reheating of the droplets, during which dendrites appear in the droplet and are translated in the graphs as noise. The solidification of a liquid is governed by heat transfer and the dynamics of the solid–liquid interface. During phase change, this interface acts as an active boundary where latent heat is released and transported through both the solid and liquid phases. When the liquid is cooled below its equilibrium solidification temperature, the process may become unstable. In such conditions, small perturbations at the interface are amplified, allowing solid protrusions to grow into the liquid and form complex microstructures.^39^ In this study, we conjecture that environmental factors, including ambient humidity, may influence dendritic growth during cooling. Nevertheless, the present experiments do not allow the individual effect of humidity to be isolated; therefore, this interpretation should be regarded as hypothetical. Although the residue of dilute sulfuric acid in the droplet may slow down the oxidation process, once the droplet is surrounded by air, the inevitable formation of the oxide layer alters the roughness of its surface. Hence, local interfacial disturbances could be introduced on the liquid metal surface that may cause moisture condensation, destabilizing the solidification front and possibly triggering dendrite formation. This explanation remains to be verified through a microstructural analysis.

**Figure 4:**
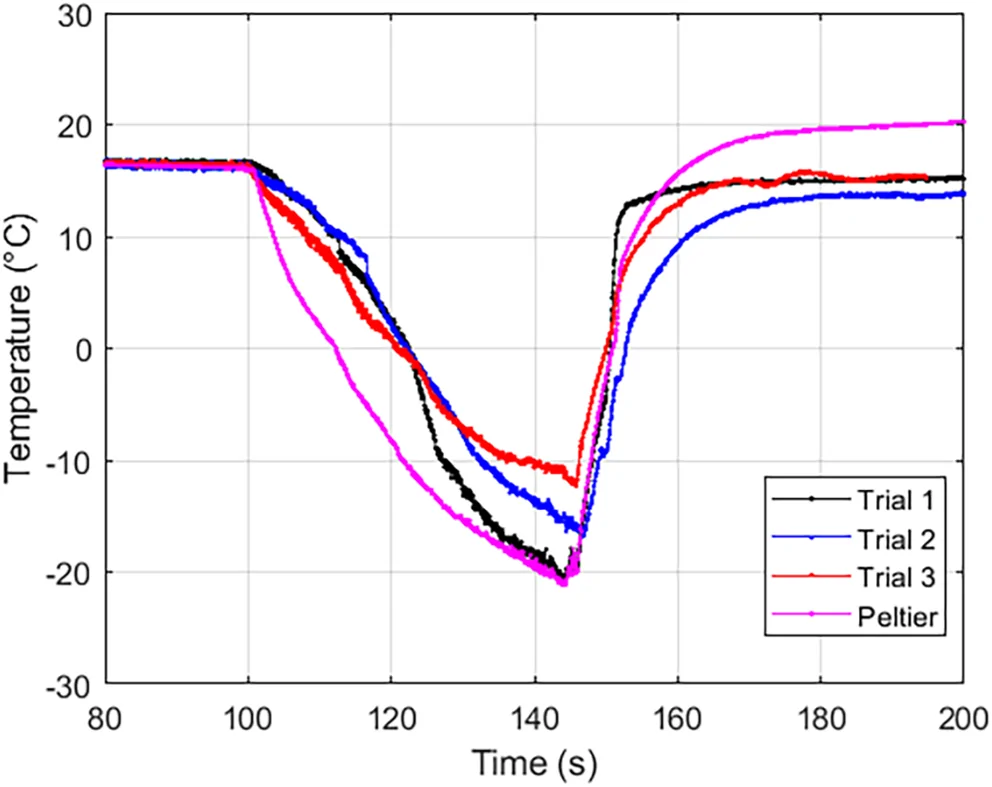
Temperature vs time for different droplets of liquid metal (Trial 1, 2, and 3) and the Peltier cell.

As the temperature rises again from its minimum, we can once more notice differences between the droplets. Some of the curves show a sharp temperature rebound, which may indicate an abrupt release of latent heat associated with a rapid phase change. Other curves exhibit a more gradual return to equilibrium, suggesting differences in thermal inertia or phase change dynamics. By the end of the recorded time (∼200 s), all samples have stabilized again around their original temperature. The differences among the curves and the presence of shoulder-like features suggest heterogeneous cooling and solidification behavior. These observations could be associated with variations in internal structure, droplet size, or surface conditions. The observed transitions could be linked to metastable states, localized nucleation, or rapid solidification phenomena that prevent complete crystallization; however, these mechanisms cannot be conclusively identified from the present data.

To provide a deeper analysis of the experimental data, we analyzed the changes in time of the temperature and volume of the droplets. To determine the droplet volume change, side-view images were processed by using a custom MATLAB script. The droplet contour was extracted from each image (Figure 5a), and a circular profile was fitted to the detected boundary points using a least-squares method (Figure 5b). Because of the high surface tension of the liquid metal, the droplet maintained a nearly spherical shape throughout the experiment, making a circular approximation of the projected profile appropriate. The radius obtained from the fitted profile was subsequently used to estimate the droplet’s volume. One limitation of the method is that the volume estimation assumes that the droplet remains approximately spherical during cooling. This assumption is supported by the observed droplet morphology and the high surface tension characteristics of liquid metals. During the dendritic growth stage, local deviations from the spherical cap geometry occur. Consequently, the circular fit method may slightly overestimate the droplet volume. However, because the dendritic protrusions occupy only a small fraction of the overall droplet size, the resulting error remains within the uncertainty bounds estimated from the radius-fitting procedure (Figure 5c). Error propagation analysis was also performed on the volume equation considering the sensitivity of the fitted radius to image resolution, which led to a 3.45–5.22% error.

**Figure 5:**
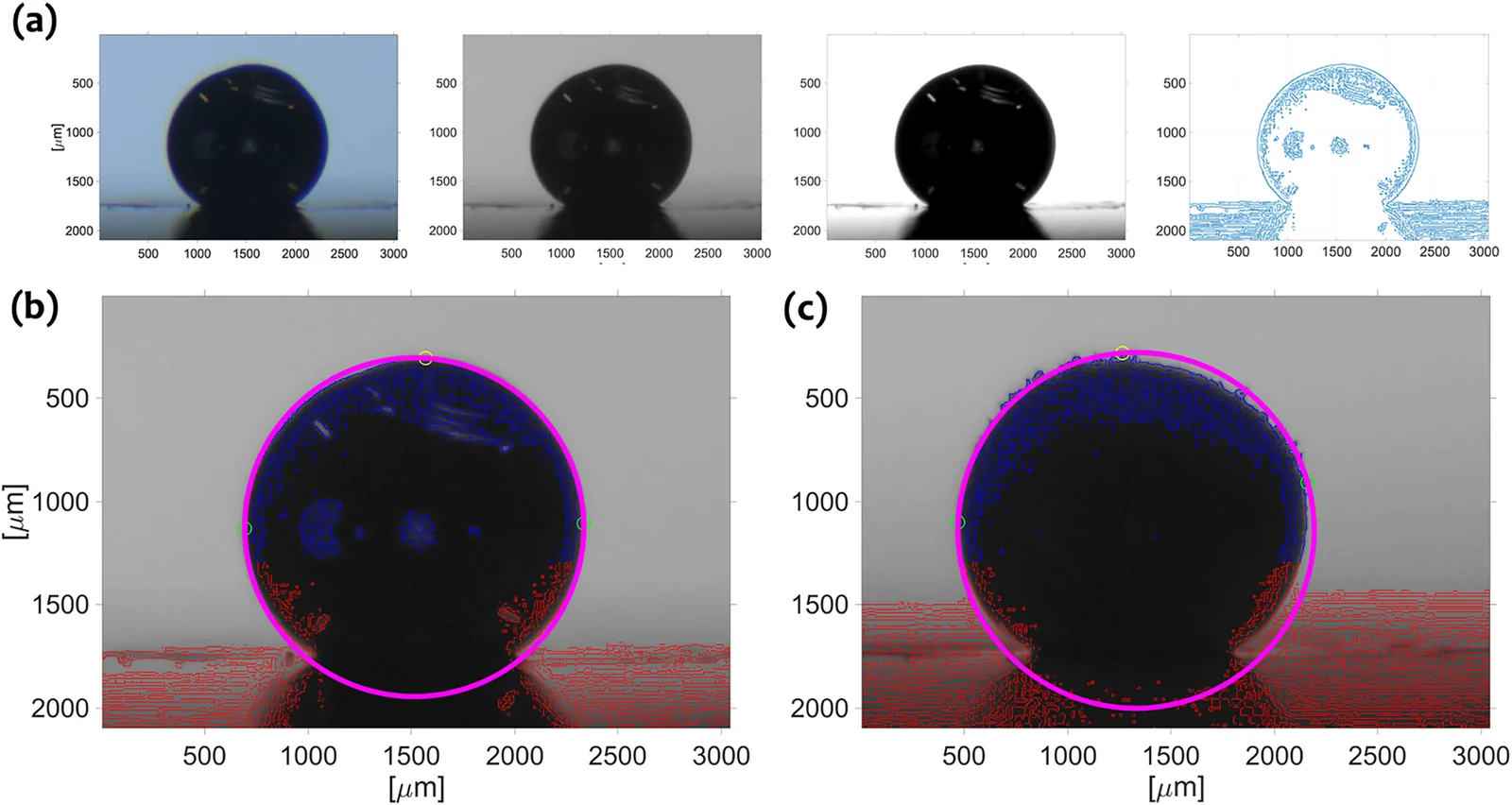
Processing sequence of a CCD image to determine the volume of the drop. (a) Conversion to grayscale, contrast enhancement and profile determination. (b) Circular fit to the droplet and (c) dendrite perturbation.

Figure 6 presents the thermal and volumetric responses of three liquid metal droplets subjected to a controlled cooling protocol. Figure 6(a) displays the temporal derivative of the temperature, Δ*T*/Δ*t*, while Figure 6(b) shows the evolution of the droplet volume. These measurements were performed simultaneously to investigate the coupling between thermal response and morphological changes during the cooling process. Figure 6(a) exhibits relatively stable Δ*T*/Δ*t* values around zero before 120 s, indicating thermal equilibrium. After approximately 100 s, a change occurs as the temperature derivatives plunge into negative values, reflecting the activation of the cooling mechanism. Several peaks emerge over time, associated with a rapid temperature recovery, likely due to the release of latent heat or a phase transition event, such as partial solidification or internal structural reconfiguration. Figure 6(b) reveals that the volume of the droplets remains constant during the initial equilibrium stage. However, around 140 s—coinciding with the thermal transitions—a noticeable increase in volume is observed. This expansion may point to structural evolution during solidification. However, because the present measurements do not provide direct evidence of the droplet’s internal morphology, alternative mechanisms—including oxide-layer deformation, morphological changes, or image-analysis uncertainties—cannot be excluded. After reaching the maximum, all curves display a volume reduction, that corresponds with the turn-off of the cooling system.

**Figure 6:**
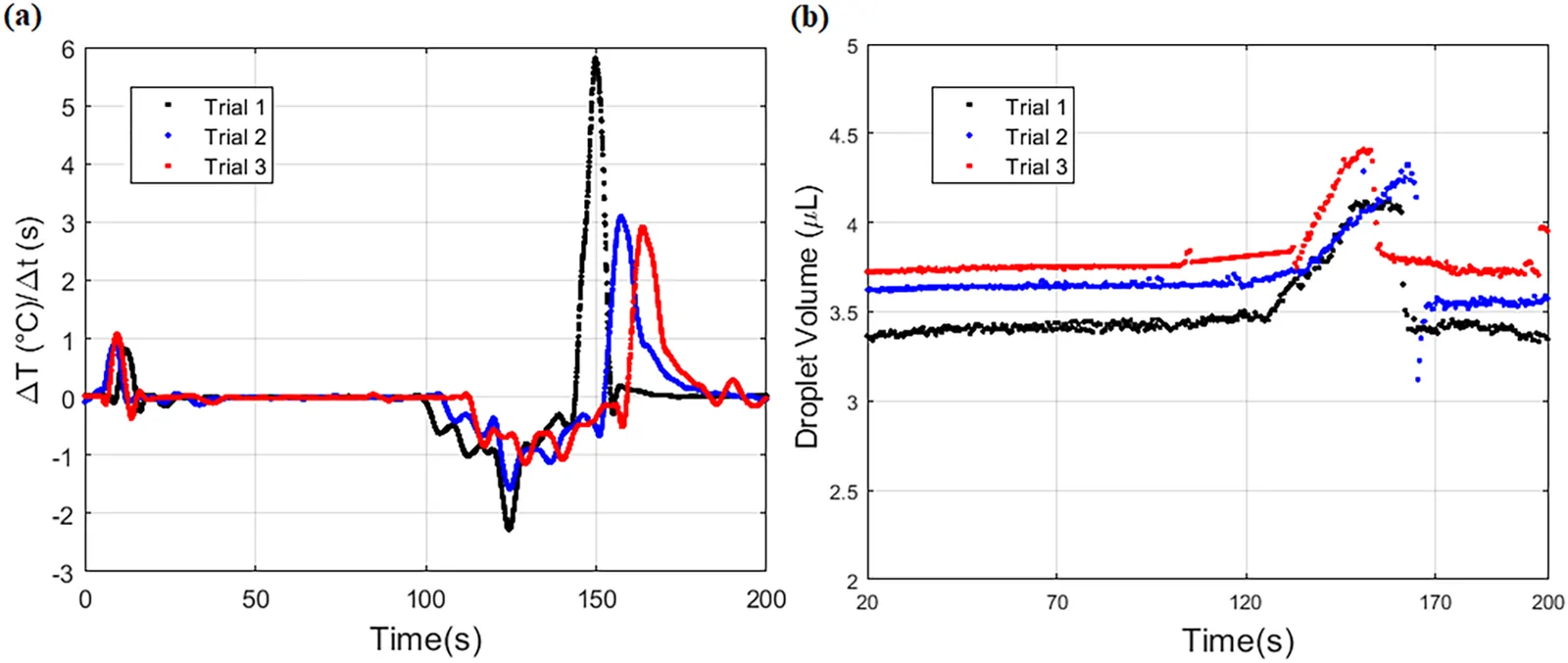
(a) Temperature rate of change (Δ*T*/Δ*t*) and (b) droplet volume as a function of time for three liquid metal samples under cooling.

These behaviors are connected through the following scaling relation, which highlights the interplay between heat extraction and the structural response of the droplet

1 $$\frac{V\Delta T}{\Delta t}=\frac{P_{T}}{\rho c_{d}}$$

where *V*(*t*) is the time-dependent volume of the droplet, Δ*T*/Δ*t* is the temperature rate of change, ρ is the density, *c_d_* is the specific heat capacity, and *P*_T_ is the thermal power delivered by the cooling system.

As we can see from Figure 6, maxima and minima appear in each experimental curve at different times. Likewise, variations in the volume (especially its increase) occur at various time values in each experiment. Therefore, normalization is necessary to determine whether these maxima and minima are characteristic of the cooling of liquid metal. Since volume appears to be the variable factor across all experiments, it is the variable that we use for normalization. To achieve this, we calculated the average volume value in three different time intervals, subsequently dividing the time values by the average volume of each droplet. Figure 7 displays the results of the time-volume normalization. The first interval spans from the beginning of the experiment until the droplet cools without the appearance of dendrites (Figure 7a). The final interval involves returning the droplet to room temperature (Figure 7b).

**Figure 7:**
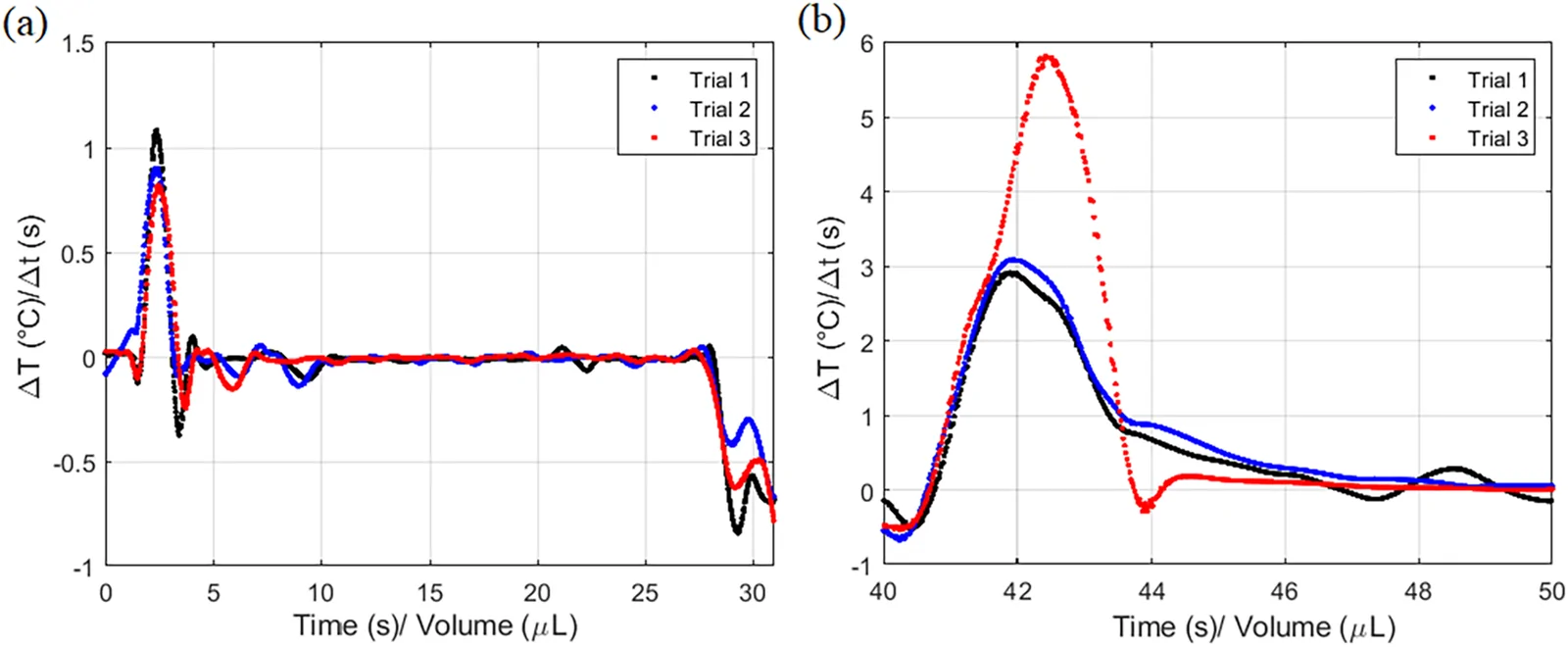
Temperature rate of change (a) droplet cooling interval, (b) heating back to ambient temperature. All the curves show the data normalized by time and volume.

As we can see, the curves match almost perfectly in the first and final sections, indicating that every transitional phase of the droplet is characteristic of liquid metal.

In turn, the intermediate interval corresponds to the appearance of the dendrites (Figure 8), which can alter the thermal behavior, as a conduction process occurs between the droplet and the environment. Even though we worked in a range-controlled humidity, this factor may represent one possible contributing influence on the differences shown in Figure 8.

**Figure 8:**
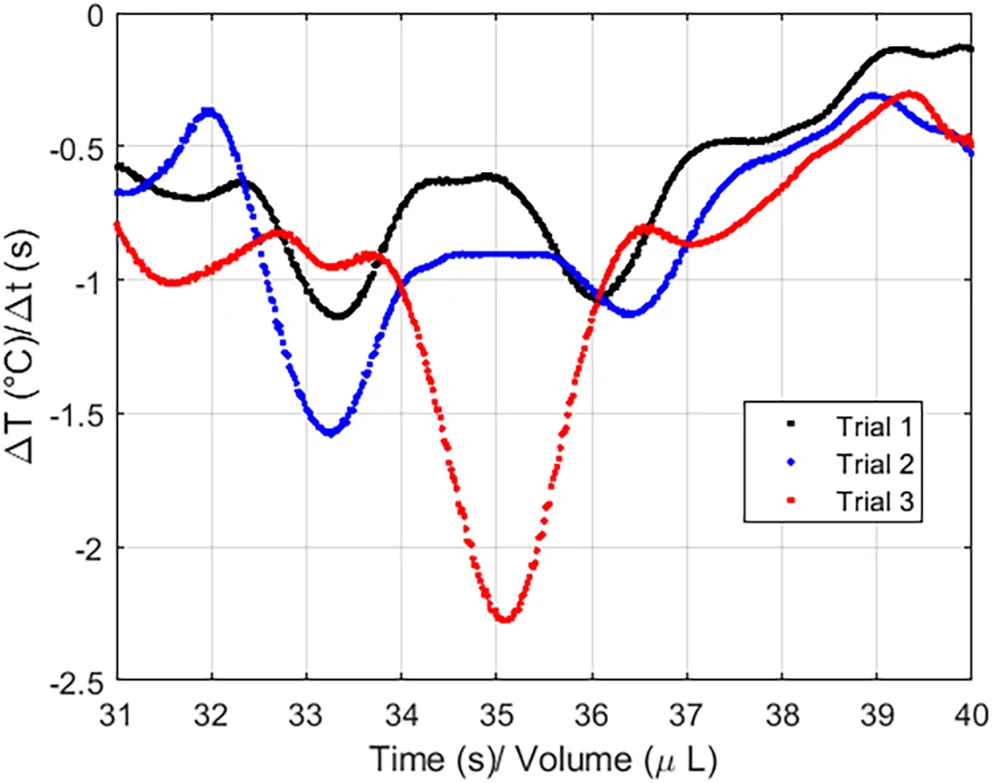
Temperature rate during dendrites appearance time interval normalized by time and volume.

From the normalized plots, we can observe characteristic peaks from the cooling and heating processes, which can be easily associated with specific temperatures. These temperatures are 10, 0, −1, and −7 °C for the cooling part and −18, −10, 0 °C for the heating part. In ref 34, a complete study of metal droplets solidification is presented and gives an insight into what happens at those temperatures. They observed a temperature hysteresis of approximately 20 °C, with galinstan solidifying near −10 °C and melting around 10 °C. Their rheological measurements showed a sharp increase in viscosity below −7 °C, indicating the onset of solidification. While the differential Scanning Calorimetry (DSC) identified an exothermic peak at −1 °C during cooling, only a single endothermic peak was observed around 10–11 °C during heating, corresponding to melting. Additionally, their wide-angle X-ray Scattering (WAXS) study confirmed the formation of crystalline phases below −10 °C, which may explain the appearance of dendritic structures in the present study at lower temperatures; nevertheless, in the absence of direct structural characterization, this connection remains speculative. The disappearance of these structures upon reheating to approximately 10 °C, further supports the rheological and thermal findings by Koh et al.^34^ Although the findings of the latter study point in the same direction as our results, demonstrating complex solidification behavior in Galinstan, it is important to note that the solidification of Galinstan was analyzed under conditions of confinement within channels or as dispersed droplets, whereas the complexity we observed in our experiments occurred in millimeter-sized droplets surrounded by air on a cold plate. In our case, we inferred the transitions from the temperature curves.

The evolution of the droplet contact angle was also measured throughout different stages of the process. In particular, angles associated with the characteristic events identified in the temporal derivative curve (Figure 9a) were measured. During the dendritic growth stage, several local fluctuations appeared in the derivative signal due to the continuous evolution of the droplet’s morphology and volume. To avoid overrepresenting this stage, only the most representative peaks were selected for the contact angle analysis. The contact angles were determined from the recorded images using the ImageJ plug-in drop analysis (dropSnake). For each selected event, five independent measurements were performed, and the reported values correspond to the mean contact angle, while the error bars represent the standard deviations of these measurements.

**Figure 9:**
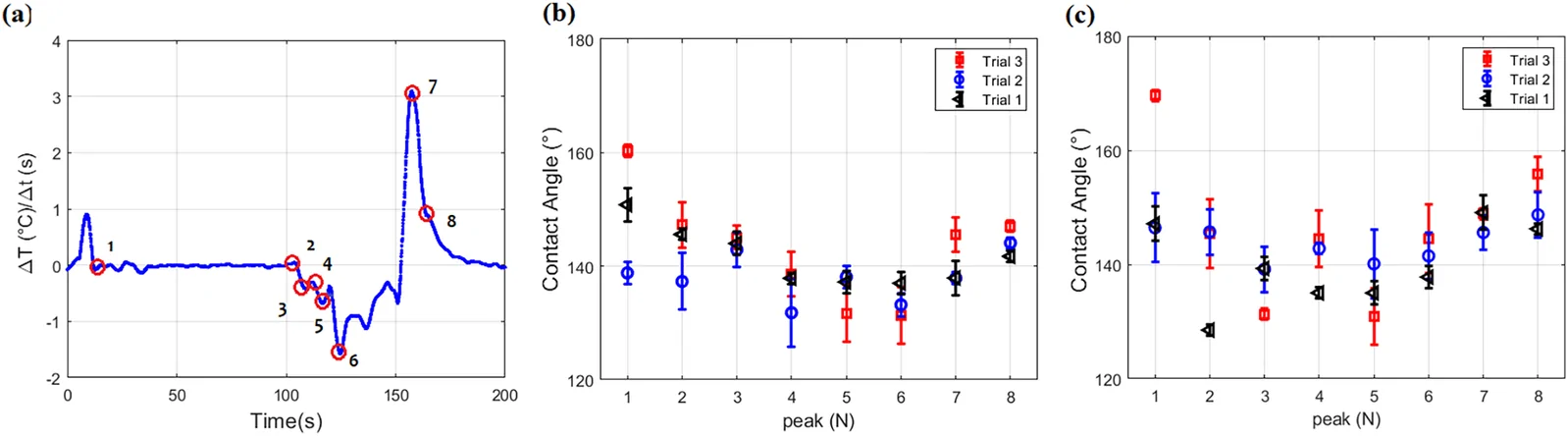
(a) Normalized temporal derivative of the droplet temperature during cooling. The numbered peaks correspond to characteristic thermal events selected for wetting analysis. In (b, c), evolution of the left and right contact angles measured at each thermal event for three independent experiments.

The resulting left and right contact angles are presented in Figure 9b,c, respectively. In general, both contact angles exhibit variations throughout the cooling process, indicating changes in the wetting behavior of the liquid metal (LM) droplet as solidification progresses. A gradual reduction of the left contact angle is observed during the intermediate stages of cooling, reaching minimum values near events 4–6. This behavior suggests modifications in the interfacial properties of the LM, such as changes in viscosity, surface tension, oxide-layer characteristics, or the appearance of transient solid phases. This behavior coincides with the abrupt thermal response observed in the derivative curve and is consistent with the possibility of a significant structural transformation occurring during this stage, although such a transformation cannot be directly confirmed from the present measurements.

Following the event (peak 7), both contact angles increase, indicating a partial recovery of the droplet shape and a modification of the balance between the surface and interfacial energies. This behavior coincides with the abrupt thermal response observed in the derivative curve and supports the interpretation that a significant structural transformation occurs during this stage. Although the overall trends of the left- and right-contact angles are similar, an asymmetry between both sides is evident. The right contact angle exhibits a larger scatter and less pronounced trends than the left contact angle. This asymmetry is likely associated with small deviations during droplet deposition, slight inhomogeneities in the aluminum substrate, or local variations in the oxide skin covering the liquid-metal surface. Since the droplet was deposited manually, a perfectly axisymmetric initial condition cannot be guaranteed, which may contribute to the observed differences between both contact angles.

This analysis is consistent with the observations of Joshipura et al.,^31^ who reported that the presence of the oxide layer affects the mechanical properties of the droplet.

## 4 Conclusions

In this work, using thermographic and optical imaging techniques, we experimentally studied the dynamic thermal behavior of cooling liquid-metal droplets. In particular, we highlight the critical and complex behavior that arises during the cooling stage, which may influence potential applications of liquid metals in areas, such as electronics and microfluidics. Additionally, the results are consistent with previously reported anomalous thermophysical properties of galinstan, including density anomalies during freezing and pronounced supercooling, thereby reinforcing its potential for thermally tunable systems.

The results demonstrate that the system does not follow a simple liquid-to-solid transformation. The observed thermal perturbations may indicate the presence of intermediate transformation processes during the cooling. Supercooling prior to crystallization supports the existence of transient states whose evolution is strongly dependent on the thermal history, material purity, and surface conditions such as oxide layer formation. Given that no direct structural characterization was performed in the present work, the existence and nature of metastable, porous, or partially crystallized phases cannot be confirmed and remain subjects of future investigation.

Analysis of the droplet revealed a gradual reduction in wetting angles during intermediate cooling stages, reaching minimum values near the minimum cooling temperature. This behavior underscores changes in interfacial properties—such as viscosity, surface tension, and oxide-skin evolution—that may be associated with intermediate phase transformations. Because the present study does not allow these effects to be independently verified, their proposed role remains hypothetical. Following significant structural transitions, when the cooling system was off, an increase in contact angles indicates a shape recovery driven by a rebalancing of surface and interfacial energies.

The preferential appearance of dendrites on the upper portion of the droplet may be related to local thermal gradients and to the interaction of the oxide-coated liquid metal surface with ambient oxygen and humidity. However, the present experiments do not permit the dominant mechanism to be identified unambiguously.

The combined analysis of temperature rate, Δ*T*/Δ*t*, and droplet volume provides valuable insight into the transient states of liquid metal under fast cooling conditions. Thermography proved to be an effective tool, not only for visualizing the cooling process in real-time but also for identifying distinct thermal events associated with phase changes, utilizing inflection points, shoulders, and noise in temperature curves. It was shown that these features in the thermal curves correlate well with physical changes in the droplet, such as dendrite formation and volume expansion associated with intermediate structures. A key distinction of this study is that all experiments were conducted successfully under ambient humidity and environmental conditions. While existing literature often explores these phase boundaries under strict confinement or within highly sophisticated vacuum environments, our findings demonstrate that complex phase tracking is achievable in open-air settings. The methodology used in this study is straightforward and replicable, relying on widely available imaging tools and standard cooling equipment. This simplicity makes it attractive for studies of other materials that undergo temperature-driven phase transitions. These results highlight the capability of thermography to detect and characterize phase transitions in dynamic, temperature-dependent media.

## Footnotes

1 Here we use the concept of metastable intermediate structures to name possible porous or foamlike structures that we cannot determine with precision from our imaging and thermographic resolutions.

## §

Centro de Estudios Mexicanos Reino Unido, Universidad Nacional Autónoma de México, Waterloo Bridge Wing 47, Room 4.13, SE1 9NH London, United Kingdom
